# The Importance of Gestation-Adjusted Birthweight Centile in Assessment of Fetal Growth in Metabolic Conditions

**DOI:** 10.4274/jcrpe.5308

**Published:** 2018-07-31

**Authors:** Caroline Ovadia, Hanns-Ulrich Marschall, Catherine Williamson

**Affiliations:** 1 King’s College London, Department of Women and Children’s Health, London, United Kingdom; 2 University of Gothenburg, Department of Molecular and Clinical Medicine, Gothenburg, Sweden

**Keywords:** Intrahepatic, cholestasis, pregnancy, birthweight, centile

## Dear Editor,

While we welcome the intention of Li et al. (1) to assess the effect of intrahepatic cholestasis of pregnancy (ICP) upon newborn birthweight, we have concerns about the authors’ interpretation of their findings because the birthweights in their meta-analysis were not corrected for gestational week or reported as a customised birthweight centile.

ICP is associated with delivery at an earlier gestational age (2), and this is reported in all of the cited manuscripts contributing to this meta-analysis (3,4,5,6,7). Using the data presented in Table 2 from the manuscript, the mean difference in gestational week of birth for the combined patient cohort of these 5 studies is 1.3 weeks (ICP 37.6±1.9, control 38.9±1.6) (1). The authors report a mean difference in birthweight of 175 g (95% confidence interval 48-301) between ICP and control babies. Between 36 and 40 weeks’ gestation, the average weekly increase in birthweight typically exceeds this figuret (8), as shown by the Canadian population data which indicates that for female fetuses the average weekly weight gain is 183 g, whilst for male fetuses the weekly weight gain is 195.75 g.

An accurate birthweight centile should be adjusted taking into consideration the baby’s sex, maternal height, weight, parity and ethnic group (9). These data were not reported in the meta-analysis by Li et al (1). However, the study by Martineau et al(6) reports customised birthweight centiles in ICP compared to normal pregnancies. This would be consistent with the reported increased ponderal index of ICP babies reported in the cited manuscript of Cheng et al(4). Thus, despite a lower absolute birthweight (at earlier gestations), the babies of ICP mothers in this study were unlikely to have features of intrauterine growth restriction.

This is consistent with multiple other studies reporting babies of ICP pregnancies to have increased birthweight centiles, including two large population cohorts reporting birthweight centiles of 6146 ICP babies compared with over 1.2 million controls (10,11); in contrast to the 198 (ICP) and 189 (control) babies reported in this meta-analysis (1).

This is of critical relevance to the management of women with ICP. The management of fetuses at risk of small for gestational age necessitates serial growth ultrasound scanning, for which there is no indication in ICP. Conversely, babies who are large for gestational age at birth have a longer-term risk of adverse metabolic health, as has been reported for babies of ICP pregnancies (12). Furthermore, the increased risk of larger fetuses in ICP will influence obstetric advice with regard to the risk of birth dystocia. It may also result in testing affected women for gestational diabetes, which is more prevalent in ICP (13).

We believe this manuscript could mislead affected women and their clinicians. Li et al’s (1) findings reflect the fact that babies born to mothers with ICP are typically of a lighter weight secondary to being born at an earlier gestation. However, most studies demonstrate that they are of a greater birth weight centile for gestational week at delivery.
